# The risk of active tuberculosis among individuals living in tuberculosis-affected households in the Republic of Korea, 2015

**DOI:** 10.1371/journal.pone.0225744

**Published:** 2019-12-17

**Authors:** Jiyeon Yang, Sodam Lee, Suhyeon Oh, Sunmi Han, Shin Young Park, Youngman Kim, Jieun Kim, Mi-sun Park

**Affiliations:** Korea Centers for Disease Control & Prevention, Cheongju, Republic of Korea; University of Otago, NEW ZEALAND

## Abstract

**Background:**

In the Republic of Korea (ROK), compared to other high-income countries, tuberculosis (TB) prevalence is relatively high. Active TB and latent TB infection (LTBI) surveillance of individuals living in TB-affected households has been conducted for several years. Although active case finding is an important strategy in low-prevalence, high-income countries, its effectiveness in a high prevalence setting is unclear. This study evaluated the risk of TB in household contact by calculating the incidence of TB among household contacts and comparing it with the general population of the ROK.

**Methods:**

A retrospective cohort study, including 36,133 household-contacts of 17,958 TB patients reported in 2015, was conducted. The data was extracted from the Korean National TB Surveillance System (web-based TB cases notification system, KNTSS). The Cox proportional hazard regression model was used to evaluate risk factors for incidence of TB. A *P*-value < .05 was considered statistically significant.

**Results:**

In this study, 319 (0.9%) of 36,133 household-contacts were reported as having TB within 1 year, which is a higher rate than the rate for the general population in the ROK. The rate of TB reported for contacts that had completed LTBI treatment (0.6%) was lower than for the LTBI group without treatment (4.6%). In multivariate analysis, age older than 65 (*p* < .001), being a spouse of a TB patient (*p* = .007), and LTBI without treatment (*p* = .013) were each a risk factor for TB incidence among contacts. Younger age (*p* < .001), presence of a cough (*p* < .001), testing positive for acid-fast bacilli (AFB; *p* < .001), and cavity on radiograph (*p* < .001) of the index patient were also statistically significant risk factors.

**Conclusions:**

Individuals living in TB-affected households are at high risk of developing TB in the ROK and active case finding among them is a strategy effective in the early detection and prevention of TB.

## Introduction

Tuberculosis (TB) is an infectious disease caused by bacteria (*Mycobacterium tuberculosis)*. TB is airborne, and its transmission rate is influenced by factors such as infectiousness of a TB patient as well as the degree of contact with a TB patient [1, 2]. Family members of TB patients are considered close contacts as they stay for a long time in the same residential area as the patient. A previous systematic literature review and meta-analysis showed that contacts of TB patients have a high-risk for developing TB, particularly within the first year of exposure [3]. Other previous studies have evaluated the effect of household contact investigation on decrease the prevalence of TB. For example, a community-randomized trial in Zambia and the Western Cape province of South Africa showed no statistically significant reduction in TB [4]. In contrast, a randomized comparative study conducted in Vietnam found that the detection rate of TB in the group that the household contact investigation was conducted, was higher than the group with the passive case finding alone among household contacts during 2-year period [5, 6]. As a result, the effect of active case finding strategy among household contacts on TB incidence remains subject to debate.

The estimated incidence and mortality rate of TB in the Republic of Korea (ROK) is 70 and 5 per 100,000 people, respectively, which is the highest among countries from the Organization for Economic Cooperation and Development (OECD) [7]. To decrease TB prevalence, the government has implemented various policies, where household contact investigation was one of the considerations [8, 9]. The ROK has conducted active TB and latent TB infection (LTBI) screening of household contacts for early detection since 2011. Nevertheless, TB incidence among household contacts in the ROK lacks sufficiently accurate estimates across the country [10–13]. This study aimed to evaluate the effect of household contact investigation on the spread of TB.

## Methods and material

### Study design and participants

This study is a retrospective cohort study conducted for an average of three years from January 2015 to July 2018. Participants were included if they were a household contact of a respiratory TB patient reported from January 2015 to December 2015 across the ROK, living in the same residential area within 3 months from the TB patient’s treatment start. To address potential sources of bias, contacts that had previously been diagnosed with TB were excluded. Additionally, TB cases reported within the first 3 months after exposure, when the base line contact investigation is conducted, were defined as ‘co-prevalent case’ and excluded in analyze for identifying the risk factors [3]. We followed the notified TB cases to establish whether the participants developed TB between date of exposure and July 2018.

### Setting

The ROK is a high-income country located in East Asia with a population of 51 million. Over 36,000 people were diagnosed with TB in 2017, which is a relatively high incidence for a high-income country [7].

It is a legal obligation to report TB cases in the ROK. When a doctor diagnoses a patient with TB at a medical institution or a public health center, a report is immediately sent to a health authority through the computerized system. Trained healthcare workers, such as staff at the public health center or nurses at the private-public mix (PPM) medical institutions, make a list of household contact persons of the reported respiratory TB patient (index patient). A household contact is defined as the person living in the same residential area as the index patient within 3 months from the index patient starting treatment. Subsequently, a screening chest X-ray or sputum test for TB and a tuberculin skin or a QuantiFERON-TB Gold In-tube test for LTBI is conducted on the contacts. The details of these procedures are described in the Korean National Tuberculosis Control Guidelines [14]. Data are recorded and managed through a web-based TB cases notification system of Korean Centers for Disease Control & Prevention (KCDC) called the Korean National TB Surveillance System (KNTSS).

### Variables

All the data used in this study were extracted from the KNTSS. Variables included in the analysis were the index patient’s gender, age, cough as a symptom, sputum test results and cavity on radiograph, along with the contact’s gender, age, LTBI treatment, time after exposure, time reported as TB and relationship type between contact and index patient. ‘Index patient’ was defined as the first person with confirmed TB within the household. ‘Household contact’ was defined as the person living in the same residential area as the index patient within 3 months from the index patient’s treatment start. ‘Incident TB’ was defined as a TB case reported 3 months after exposure, which was the time point when baseline household contact investigation was conducted. ‘Co-prevalent TB’ was the TB case reported within the first 3 months of exposure [3]. To confirm ‘LTBI’, a tuberculin skin test (cut-off value of 10 mm), or a QuantiFERON-TB Gold In-tube test (cut-off value of 0.35 IU/mL) was conducted. The’ LTBI treatment status’ was established using health records created by trained health staff involved in household contacts investigation and management, following the Korean National Tuberculosis Control Guidelines. ‘Time after exposure’ was defined as the period between the date when the index patient was reported and the date when the contact was reported as TB. ‘Relationship type’ was stratified to include a spouse, a first degree relative (parent, child, sibling) or a second degree relative (grandparent, grandchild, others).

### Study population

**Fig 1:**
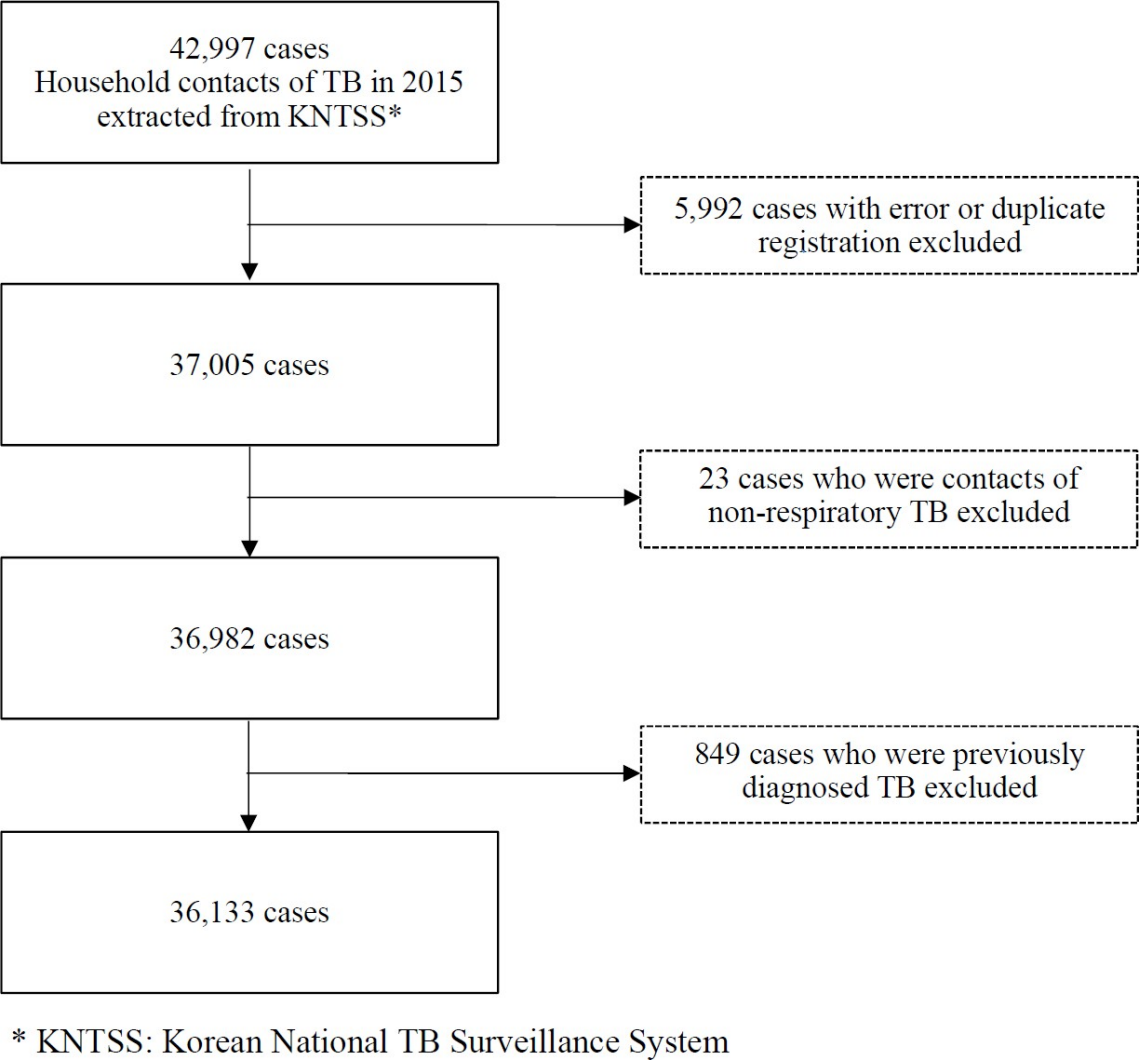
Flow diagram of study population.

Overall, 42,997 cases were extracted from the KNTSS. The following participants were excluded: 5,992 cases with error or duplicate registration, 23 contacts of non-respiratory TB, 849 cases previously diagnosed TB. The final sample included 36,133 (Fig 1). This study was conducted in accordance with Korean Infectious Disease Control and Prevention Act and Tuberculosis Prevention Act with permission of KCDC. The study design was approved by the Institutional Review Board of KCDC.

### Statistical analysis

The demographic and clinical characteristics of the index patients and their contacts were described with counts, rates, means and standard deviation. TB cases according to time after exposure of the household contacts were reported as cumulative counts and rates. Cox regression analysis was used to identify the risk of TB based on contacts’ and patents’ characteristics and reported as hazard ratios and corresponding 95% confidence intervals (CI). To identify risk factors associated with incident TB cases among household contacts, multivariate analysis was performed with variables determined as significant in univariate analysis (*p*-value < .05) and variables found clinically important in previous studies. Wilcoxon method was used to estimate risk of TB based on LTBI treatment. Statistical analysis was performed using SAS version 9.4(SAS Institute Inc). *P*-value < .05 was considered statistically significant.

## Results

Table 1 summarizes the demographic and clinical characteristics of 17,958 index patients. The mean age of the patients was 55.2-year-old, and 61.9% were male. In addition, 40.9% of the patients were positive for acid-fast bacilli (AFB); cavitation was observed on chest X-ray in 24.7% of cases.

**Table 1:** Demographic and clinical characteristics of the index cases.

Characteristics	Index cases	
	Number	(%)
**Total**	**17,958**	(100.0)
Gender		
Male	11,120	(61.9)
Female	6,838	(38.1)
Age (year, mean ± SD)	(55.2±20.4)	
0–18	644	(3.6)
19–64	10,395	(57.9)
≥ 65	6,919	(38.5)
Cough as a symptom		
Yes	9,818	(54.7)
No	8,140	(45.3)
Sputum test result		
Smear-positive	7,340	(40.9)
Smear-negative	9,684	(53.9)
Unknown	934	(5.2)
Cavity on radiograph		
Yes	4,433	(24.7)
No	12,973	(72.2)
Unknown	552	(3.1)

The demographic and clinical characteristics of 36,133 contacts are presented in Table 2. The average age of the contacts was 40.6 years, and 58.6% were female. First-degree relatives constituted 53.6% of the contacts, followed by spouses (29.9%), and second-degree relatives (16.5%). Among the subjects, 37.5% (13,551/36,133) were tested for LTBI; 24.9% were identified as having LTBI.

**Table 2:** Demographic and clinical characteristics of index cases’ household contacts.

Characteristics	Household contacts	
	Number	(%)
**Total**	**36,133**	**(100.0)**
Gender		
Male	14,961	(41.4)
Female	21,172	(58.6)
Age (year, mean ± SD)	(40.6±22.5)	
0–18	7,994	(22.1)
19–64	22,419	(62.1)
≥ 65	5,720	(15.8)
LTBI*		
LTBI and therapy Complete	683	(1.9)
LTBI and therapy started but not Complete	1,050	(2.9)
LTBI and no therapy	1,635	(4.5)
No LTBI	10,183	(28.2)
Not tested	22,582	(62.5)
Relationship type^†^		
Spouse	10,812	(29.9)
First degree relatives	19,369	(53.6)
Second degree relatives	5,952	(16.5)

* Treatment status based on records created by trained health staff involved in household contacts investigation and management, following the Korean National Tuberculosis Control Guidelines.

† First degree relatives: parent, child, sibling; Second degree relatives: grandparent, grandchild, others

A total of 36,133 household contacts of TB patients reported in 2015 in the ROK were followed up until July 2018. A total of 512 contacts (1.4%) were reported to be TB patients. The cumulative incidence rate was 186 (0.5%) for 3 months, 237 (0.7%) for 6 months, 319 (0.9%) for 1 year, and 407 (1.1%) for 2 years (Table 3).

**Table 3:** Number of TB cases according to time after exposure of the household contacts.

Time after exposure*	≤3 months^†^	≤6 months	≤9 months	≤1 year^‡^	≤2 years	Total
Reported TB cases (%)	186	237	280	319	407	512
	(0.5)	(0.7)	(0.8)	(0.9)	(1.1)	(1.4)

* Time after exposure defined as the period between the date when the index patient was reported and the date when the contact was reported as TB

† 1 month = 30 days

‡ 1 year = 365 days

**Fig 2:**
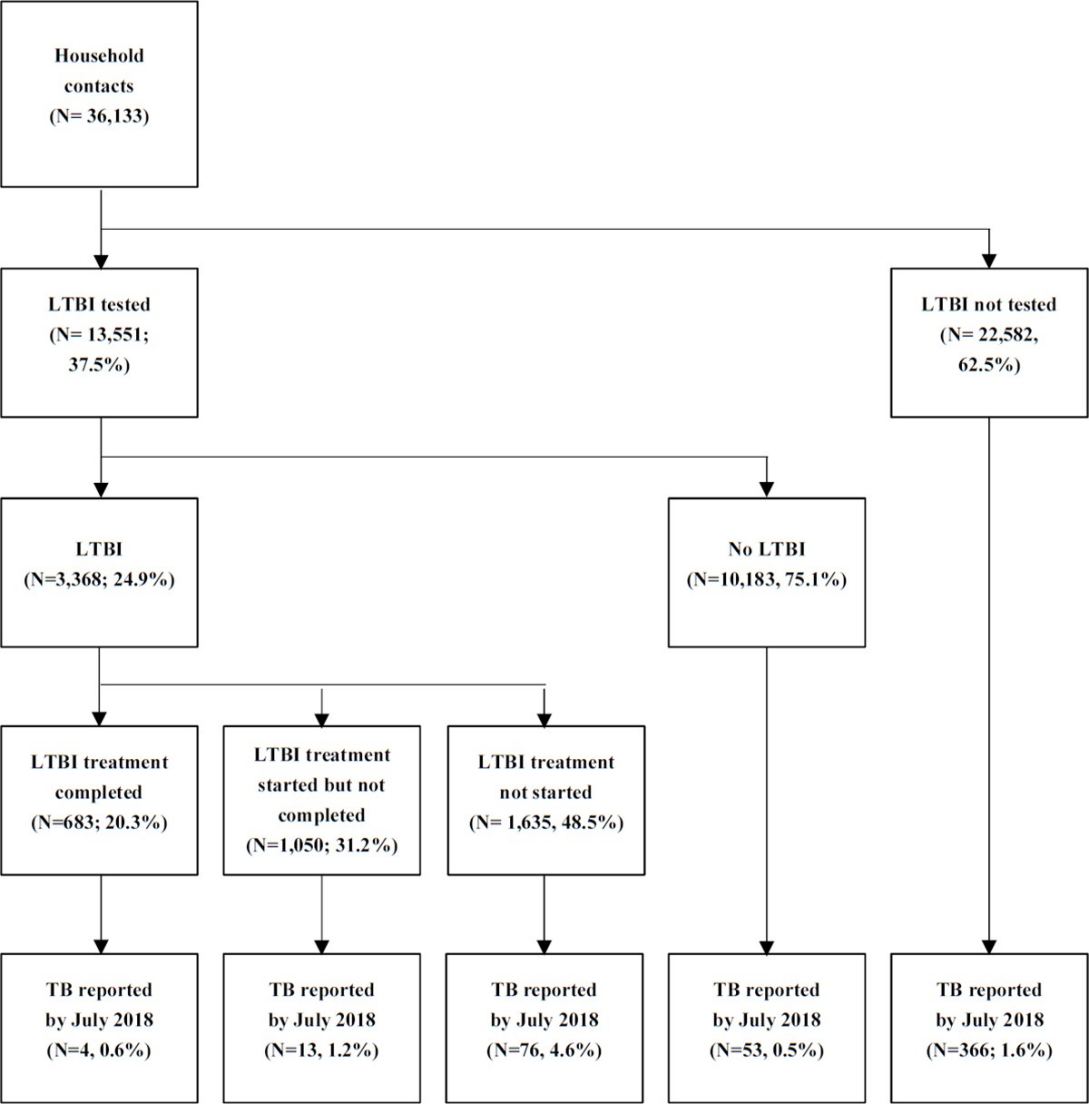
Flow diagram of newly developed TB case among household contacts by LTBI treatment.

**Fig 3:**
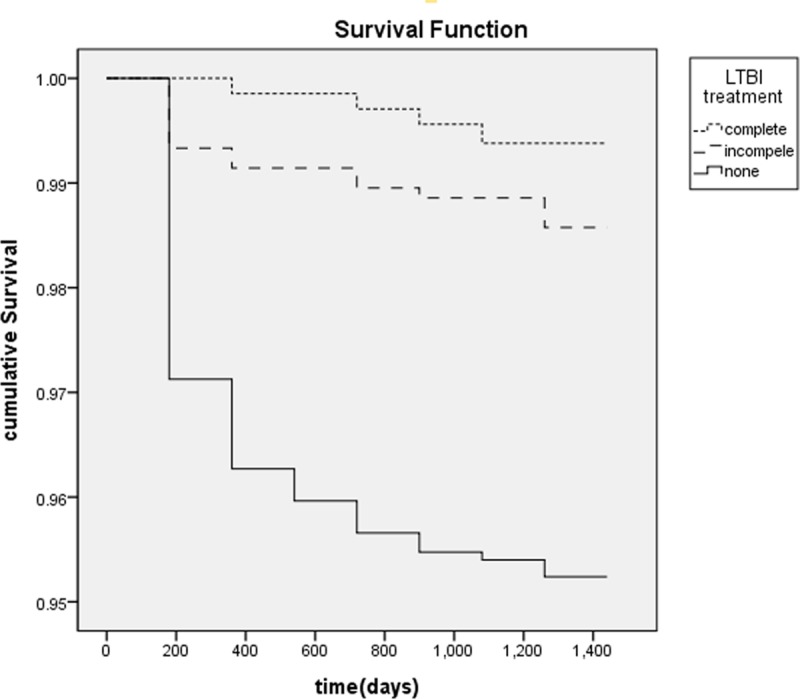
Survival function of TB by Gehan’s Wilcoxon method depending on LTBI treatment (p < .001).

The incidence and survival function of TB according to the treatment of LTBI is shown in Figs 2 and 3, respectively. Among the contacts with LTBI, 0.6% (4/683), 1.2% (13/1,050), and 4.6% (76/1,635) developed TB in the treatment-completed, treatment-not-completed, and treatment-not-started group respectively.

Multivariate analysis identified significant risk factors for incidence of TB (Table 4). These included age 0–18 years (adjusted hazard ratio [aHR], 3.1; 95% confidence interval [CI], 1.8–5.5; p <0.001), 19–64 years (aHR, 1.8; 95% CI, 1.3–2.3; *p* <0.001), presence of a cough (aHR, 1.7; 95% CI, 1.3–2.2; *p* <0.001), positive test for AFB (aHR, 2.7; 95% CI 2.1–3.5; *p* <0.001), and cavity on radiograph (aHR, 1.6; 95% CI, 1.3–2.0; *p* <0.001) of the index patient. Risk factors associated with TB among contacts were age older than 65 years (aHR, 3.6; 95% CI, 2.2–5.8; *p* <0.001), and LTBI without treatment (aHR, 3.7; 95% CI, 1.3–10.4; *p* = 0.013). Regarding the relationship between the index patient and the contact person, having a spouse with TB increased the risk of transmission (aHR, 1.8; 95% CI, 1.2–2.8; *p* = 0.007).

**Table 4:** Factors associated with incident TB cases among household contacts.

	Contacts without incident TB		Contacts with incident TB*		Crude Hazard Ratio		Adjusted Hazard Ratio	
	Number	(%)	Number	(%)	(95% CI)	*p*-value	(95% CI)	*p*-value
**Total**	**35,621**	(100.0)	**326**	(100.0)				
Index factors								
Gender								
Male	21,051	(59.1)	218	(66.9)	1.4 (1.1–1.8)	0.005	1.2 (1.0–1.6)	0.117
Female	14,570	(40.9)	108	(33.1)	Reference		Reference	
Age								
0–18	1,537	(4.3)	17	(5.2)	1.4 (0.9–2.4)	0.185	3.1 (1.8–5.5)	<0.001
19–64	21,493	(60.3)	211	(64.7)	1.3 (1.0–1.6)	0.060	1.8 (1.3–2.3)	<0.001
≥ 65	12,591	(35.3)	98	(30.1)	Reference			
Cough as a symptom								
Yes	19,596	(55.0)	244	(74.8)	2.4 (1.9–3.1)	<0.001	1.7 (1.3–2.2)	<0.001
No	16,025	(45.0)	82	(25.2)	Reference		Reference	
Sputum test								
Smear-positive	14,978	(42.0)	233	(71.5)	3.4 (2.7–4.4)	<0.001	2.7 (2.1–3.5)	<0.001
Smear-negative	18,913	(53.1)	86	(26.4)	Reference		Reference	
Unknown	1,730	(4.9)	7	(2.1)	0.9 (0.4–1.9)	0.739	1.0 (0.4–2.1)	0.903
Cavity on radiograph								
Yes	8,899	(25.0)	149	(45.7)	2.5 (2.0–3.1)	< .001	1.6 (1.3–2.0)	<0.001
No	25,699	(72.1)	171	(52.5)	Reference		Reference	
Unknown	1,023	(1.8)	6	(1.8)	0.9 (0.4–1.9)	0.698	0.9 (0.4–2.0)	0.744
Contact factors								
Gender								
Male	14,734	(41.4)	136	(41.7)	1.0 (0.8–1.3)	0.892	1.3 (1.0–1.6)	0.064
Female	20,887	(58.6)	190	(58.3)	Reference		Reference	
Age								
0–18	7,922	(22.2)	37	(11.3)	Reference		Reference	
19–64	22,132	(62.1)	189	(58.0)	1.8 (1.3–2.6)	0.001	1.5 (1.0–2.2)	0.073
≥ 65	5,567	(15.6)	100	(30.7)	3.8 (2.6–5.6)	< .001	3.6 (2.2–5.8)	<0.001
LTBI^†^								
LTBI and treatment Complete	679	(1.9)	4	(1.2)	Reference		Reference	
LTBI and treatment started but not Complete	1,037	(2.9)	8	(2.5)	1.3 (0.4–4.4)	0.651	1.4 (0.4–4.7)	0.566
LTBI and no treatment	1,559	(4.4)	38	(11.7)	4.1 (1.5–11.5)	0.007	3.7 (1.3–10.4)	0.013
No LTBI	10,130	(28.4)	41	(12.6)	0.7 (0.3–1.9)	0.482	0.9 (0.3–2.5)	0.827
Not tested	22,216	(62.4)	235	(72.1)	1.8 (0.7–4.8)	0.248	1.5 (0.6–4.3)	0.407
Relationship type^‡^								
Spouse	10,621	(29.8)	135	(41.4)	2.6 (1.8–4.0)	<0.001	1.8 (1.2–2.8)	0.007
First degree relatives	19,100	(53.6)	163	(50.0)	1.8 (1.2–2.7)	0.005	1.5 (1.0–2.2)	0.075
Second degree relatives	5,900	(16.6)	28	(8.6)	Reference			

* TB cases within 90 days from diagnosis of index patients were defined as ‘co-prevalent TB’ and excluded in risk analysis.

† Treatment status based on records created by trained health staff involved in household contacts investigation and management, following the Korean National Tuberculosis Control Guidelines

‡ First degree relatives: parent, child, sibling. Second degree relatives are grandparent, grandchild, others.

## Discussion

In this study, a total of 1.4% of family contacts were reported as having TB within the follow-up period (average of 3 years); overall, 24.9% were confirmed as LTBI through the household contact investigation. According to a systematic literature review and meta-analysis, in high-income countries, TB prevalence among contacts is estimated at 1.4% with the rate of LTBI estimated at 28.1% [3], which is similar to the results of the present study. This study also shows that the incidence of TB among the Korean household contacts is higher than that of the general population. The 1-year incidence rate in this study was 0.9%, which corresponds to 883 persons per 100,000 people; this is much higher than the estimated incidence across the ROK reported as 70 per 100,000 people [7, 15]. Family members of TB patients are at high risk of TB due to close contact with patients, high frequency of exposure, and long exposure period. This suggests family contacts are an important element of TB control and management in the ROK, which is consistent with some of previous studies in high prevalence setting such as Vietnam or India [5, 6, 16].

The incidence of TB was high within the first 3 months from the index patient report, which may have resulted from early detection through active contact screening at the time of index case TB reporting. Despite early detection, during the follow-up period, the incidence rate among the contacts remained higher than among the general population, suggesting the need for follow-up TB screening among the contacts [16–18].

In this study, among the people with LTBI and without preventive therapy, 4.6% progressed to TB, which is consistent with previous studies estimating that untreated LTBI turns into, TB in 5 to 10% of cases, with majority of new cases presenting within the first few years of infection [7, 19, 20]. Furthermore, in our study the risk of TB in the untreated LTBI group (aHR, 3.7; 95% CI, 1.3 to 10.4; *p* = .013) was significantly higher than in the treatment completed LTBI group.

Among the participants, the LTBI examination rate and the LTBI treatment completion rate were 37.5% and 20.3%, respectively. The treatment completion rate was lower than the equivalent in the United States (58%) and much lower than the equivalent in Japan, where reporting of LTBI is mandatory, and where treatment completion is estimated at 71.9% [21,22]. The low rate in our study might be due to low awareness of the importance of TB contact examination and treatment even among contacts that understand they had been exposed to TB [11]. Another possible explanation for these findings might be that the national policy at the time, required examination or treatment only of household contacts that were under-35 years of age or high-risk of TB [14]. Following the World Health Organization’s guidelines emphasizing the importance of treatment for LTBI, the ROK has recently begun actively screening for LTBI and offering treatment for LTBI to household contacts regardless of age. Further education of medical staff (in case of the ROK, at a PPM medical institution or public health center) is nevertheless required to improve LTBI testing and treatment rate, as nurses and doctors have opportunities to enhance awareness of the importance of testing, build trust in the preventive therapy and help household contacts complete the treatment safely. However, further studies are needed to identify barriers to LTBI treatment and improve drug compliance according to cultural and social background, which has been suggested as a determinant [21,22,23,24].

The relationship between the index patient and the contact person also affected the risk of TB transmission in this study. For spouses, the hazard ratio was 1.8 (*p* = .007) compared with that of the second-degree relatives (grandparent, grandchild, others). This is likely to result from a spouse maintaining a closer contact with the index patient, which usually includes sharing the bedroom [13,17].

Our study has some limitations. Although a significant number of TB cases was confirmed within the first 3 months from exposure (contact investigation), these immediate TB cases were excluded from the analysis of risk factor as it was not possible to verify the source of infection [3, 13]. Moreover, as this was a retrospective study it was difficult to investigate other risk factors. For example, nutrition or immune status of household contacts could not have been considered in our study [1, 9, 13, 20].

## Conclusion

This study showed the incidence of TB was higher among individuals living in TB-affected households than among the general population. For index patients, younger age, cough as a symptom, positive AFB, and cavity on radiograph were statistically significant risk factors for TB transmission in household contacts. The risk factors related to contacts were older age, having a spouse with TB, and LTBI not treated. Raising awareness of importance and effectiveness of preventive therapy for household contacts through education to public and medical staff may increase the rate of LTBI detection and treatment completion among household contacts.
